# Immunization with a Peptide Containing MHC Class I and II Epitopes Derived from the Tumor Antigen SIM2 Induces an Effective CD4 and CD8 T-Cell Response

**DOI:** 10.1371/journal.pone.0093231

**Published:** 2014-04-01

**Authors:** Haydn T. Kissick, Martin G. Sanda, Laura K. Dunn, Mohamed S. Arredouani

**Affiliations:** 1 Department of Surgery, Urology Division, Beth Israel Deaconess Medical Center, Harvard Medical School, Boston, Massachusetts, United States of America; 2 Department of Urology, Emory University School of Medicine, Atlanta, Georgia, United States of America; Leiden University Medical Center, Netherlands

## Abstract

Here, we sought to determine whether peptide vaccines designed harbor both class I as well as class II restricted antigenic motifs could concurrently induce CD4 and CD8 T cell activation against autologous tumor antigens. Based on our prior genome-wide interrogation of human prostate cancer tissues to identify genes over-expressed in cancer and absent in the periphery, we targeted SIM2 as a prototype autologous tumor antigen for these studies. Using humanized transgenic mice we found that the 9aa HLA-A*0201 epitope, SIM2_237–245_, was effective at inducing an antigen specific response against SIM2-expressing prostate cancer cell line, PC3. Immunization with a multi-epitope peptide harboring both MHC-I and MHC-II restricted epitopes induced an IFN-γ response in CD8 T cells to the HLA-A*0201-restricted SIM2_237–245_ epitope, and an IL-2 response by CD4 T cells to the SIM2_240–254_ epitope. This peptide was also effective at inducing CD8^+^ T-cells that responded specifically to SIM2-expressing tumor cells. Collectively, the data presented in this study suggest that a single peptide containing multiple SIM2 epitopes can be used to induce both a CD4 and CD8 T cell response, providing a peptide-based vaccine formulation for potential use in immunotherapy of various cancers.

## Introduction

Defined epitope vaccines elicit an immune response by immunization with a synthetic fragment derived from the target protein. This synthetic fragment is most commonly a 9–10aa long peptide selected to bind human leukocyte antigen (HLA) class I. In the case of cancer vaccines, epitopes that are restricted to a particular MHC-I haplotype are designed and used to stimulate the immune system against tumor-associated antigens (TAAs) [1]. In recent years, this approach for vaccine development has delivered many immunogenic epitopes derived from known TAAs [1]–[4]. With the advent of high throughput methodologies, the TAA-derived immunogenic epitope portfolio has been significantly enriched due to comprehensive profiling of TAAs of all cancer types.

Peptide-based vaccines for cancer therapy have been developed and subjected to preclinical and clinical testing in numerous studies. Most notably, vaccination with the gp100-209:217(210M), an HLA-A*0201-restricted epitope derived from the melanoma antigen gp100, significantly improved the clinical response and median overall survival of stage IV melanoma patients receiving IL-2 therapy [2]. While peptide-based cancer vaccines had limited success through the years, the survival benefit gained from the gp100-209:217(210M) melanoma epitope vaccine trial was received with much enthusiasm, and has reinvigorated interest in peptide vaccines for cancer immunotherapy. Clinical trials in numerous cancers including melanoma, mesothelioma, colorectal and cervical cancer have been completed and shown this could be an effective strategy for inducing a clinically beneficial immune response against TAAs [1]. Recent studies suggest the inclusion of multiple MHC class I restricted epitopes and addition of MHC class II epitopes in a single longer peptide to improve vaccine outcome [5]–[8]. Longer multi-epitope peptides targeting p53 have been shown to induce a p53-specific CD4 and CD8 T-cell response in early stage clinical trials against colorectal cancer [9]. Similarly, long peptide immunization against the mesothelioma antigen WT1 induced antigen-specific, CD4 and CD8 T cell response in 6 out of 9 patients [10]. Most impressively, a multi-epitope vaccine against the Human Papillomavirus (HPV) oncogenic E6 and E7 proteins to treat HPV-induced vulvar intraepithelial neoplasia resulted in reduction in symptoms in 60% of patients and complete clearance of disease in 25% of them [11]. These clinical findings support the idea that multi-epitope vaccines can induce effective CD4 and CD8 anti-TAA responses resulting in measurable clinical benefit.

Using a genome-wide interrogation strategy to identify genes that are expressed abundantly in human prostate cancer but sparsely in non-cancerous adult tissues, we previously identified numerous putative prostate TAAs including ETS related gene (ERG) and Single-minded homolog 2 (SIM2) [3], [4]. Additionally, we have identified SIM2-derived, HLA-A*0201–restricted, immunogenic epitopes with potential anti-cancer activity [3], [12]. Here we aimed to further investigate the immunogenicity of SIM2-derived peptides using humanized mice and human prostate HLA-A*0201-positive cell lines expressing this antigen. We also designed and tested longer peptides harboring multiple MHC-I and MHC-II-restricted epitopes to evaluate whether peptide vaccines that deliver both class-I and class-II restricted epitopes could concurrently induce CD4 and CD8 T cell activation responsiveness *in vivo* with a single peptide.

## Methods

### Mice and animal ethics statement

HHD mice were obtained from Dr. Francois Lemonnier (Unite d'Immunité Cellulaire Antivirale, Institut Pasteur, Paris, France). These mice are β_2_m−/−, Db−/− double knockout and express an HLA-A*0201 mono-chain composed of a chimeric heavy chain (α1 and α2 domains of HLA-A*0201 allele and the α3 and intracellular domains of Db allele) linked by its NH_2_ terminus to the COOH terminus of the human β2m by a 15–amino acid peptide arm [13]. All mice were housed in pathogen-free conditions, and all experimental procedures involving animals were approved by the Institutional Animal Care and Use Committee at Beth Israel Deaconess Medical Center.

### Cell line

T2 cells used in HLA-A*0201 binding assays and as targets in ELISPOT assays were obtained from ATCC and cultured as described in the accompanying product protocol. PC3 and LNCaP lines were obtained from ATCC. PC3-A*0201^+^ cells were produced by transfecting wild type PC3 cells with an HLA-A*0201-puromycin containing retrovirus produced as described in Maeurer *et al* [14]. The HLA-A*0201-containing plasmid was a gift from Dr. Gordon Freeman at Dana Farber Cancer Institute.

### *In silico* analysis of gene expression data

SIM2 gene expression data were obtained through the Oncomine Research Edition (www.oncomine.org). The database was queried for microarray datasets that show a 2-fold change in SIM2 expression and a p value<.01 between cancer and control groups.

### Peptide design

The SIM2 protein sequence was downloaded from the NCBI protein database (NP_005060.1). The IEDB (http://www.iedb.org/) epitope prediction algorithm (Available at http://tools.immuneepitope.org/main/html/tcell_tools.html) was then used to predict regions of the protein that may bind MHC-I and MHC-II molecules [15], [16].

### SIM2-derived peptide immunogenicity in transgenic mice

Eight- to 12-wk-old male HHD mice were injected sub-cutaneously on the right flank with 100 µg of each candidate peptide emulsified in 50 µL of incomplete Freund's adjuvant and 50 µL PBS in the presence of 150 µg of the I-Ab–restricted HBVcore_128–140_ T helper epitope (TPPAYRPPNAPIL) [17] or the SIM2 derived I-Ab epitope, LKLIFLDSRVTEVTG. Mice immunized with the long SIM2 peptide received 150 ug total under the same conditions. Ten to 12 d after immunization, spleens were harvested and splenocytes were tested for peptide-induced specific release of IFN-γ by enzyme-linked immunospot (ELISPOT) assay.

### ELISPOT assay

ELISPOT was performed as described by the manufacturer's instruction. Briefly, 96-well Millipore Immobilon-P plates were coated with 100 µL/well mouse IFN-γ specific capture mAb (AN18; Mabtech, Inc.) at a concentration of 10 µg/mL in PBS overnight at 4°C. To investigate the recall response to immunization with various peptides, a total of 2.5×10^5^ splenocytes were seeded in each well in four replicates, and 2.5×10^5^ peptide-loaded (10 µg peptide/mL, for 2 h at 37°C) splenocytes pretreated with 50 µg/mL mitomycin C for 1 h were added to each well. To investigate the response of immunized mice to prostate cancer cell lines, 5×10^4^ splenocytes isolated from immunized mice were cultured with 5×10^4^ tumor cells pretreated with 50 µg/ml of mitomycin C for 1 h. ELISPOT was developed as described in manufacturer's instruction (Mabtech, Murine IFN-γ ELISPOT kit). Spots measured in these experiments were multiplied by the appropriate dilution factor to express IFN-γ producing cells per million splenocytes.

To measure the IL-2 response of CD4 T-cells, pure CD4^+^ T-cells were isolated using the EasySep mouse CD4 T-cell enrichment kit from StemCell Technologies (Cat: 19752). IL-2 ELISPOT was performed as described by the manufacturer's instruction (eBioscience; 88-7824). CD4^+^ cells were co-cultured with splenocytes loaded with various peptides (10 µg peptide/mL, for 2 h at 37°C) and treated with 50 µg/mL mitomycin C for 1 hr. ELISPOT plates were developed after 24 hours.

### Intracellular flow cytometry

Splenocytes were isolated from immunized HHD mice and co-cultured at a 1∶1 ratio with T2 cells loaded with 10 µg of peptide/mL, for 2 h at 37°C. Cells were incubated overnight with Brefeldin A. Cells were stained for surface antigens and then permeabilized using eBioscience permeabilization buffers (eBioscience; 88-8824-00), and then stained intracellularly for IFN-γ. Cells was analyzed with a BeckmanCoulter Galios flow cytometer.

### Statistical analysis

Statistical analysis was performed using the Student's T-test. P values of less than 0.05 were considered significant and are denoted by an asterisk in figures.

## Results

### SIM2 is overexpressed in various cancers

**Figure 1:**
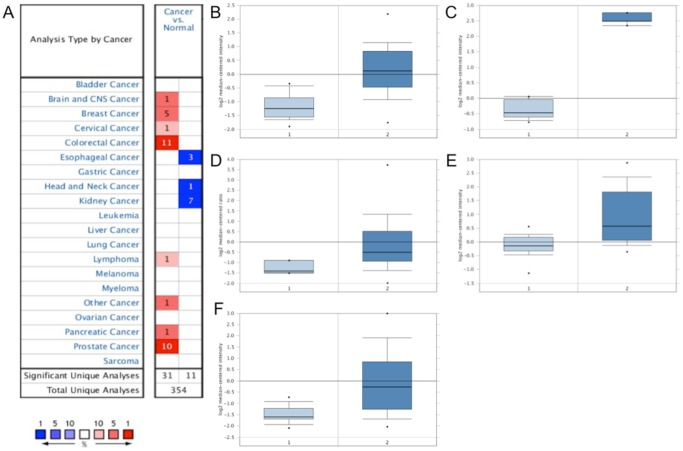
Human SIM2 gene expression analysis in various cancers. SIM2 gene expression data were extracted from the Oncomine Research Edition. Microarray datasets that show a 2-fold change in SIM2 expression between cancer and control groups and a p value<0.01 are highlighted. (**A**) Comparison of SIM2 gene expression between cancer and control specimens. Red color indicates SIM2 overexpression and the blue color indicates SIM2 down-regulation in cancer. Numbers in the boxes indicate the number of datasets showing statistical significance. Box plots were obtained from the datasets selected in (**A**) to highlight significant overexpression of SIM2 in Prostate Carcinoma (1. Prostate Gland (n = 23), 2. Prostate Carcinoma (n = 65); *P* = 2.41×10^−14^, [40]) (**B**); Colon Carcinoma (1. Colon (n = 10), 2. Colon Carcinoma (n = 5); P = 1.65×10^−12^ [41]). (**C**); Breast Carcinoma (1. Breast (n = 4), 2. Invasive Breast Carcinoma (n = 154); P = 2.25×10^−4^, [42]) (**D**); Oligodendroglioma (1. Brain (n = 23), 2. Oligodendroglioma (n = 50); P = 3.31×10^−9^ [43]) (**E**); and Pancreatic Carcinoma (1. Pancreas (n = 16), 2. Pancreatic Carcinoma (n = 36); P = 3.01×10^−7^ [44]) (**F**).

Previously, we reported that SIM2 was an ideal target for prostate cancer immunotherapy, being a protein overexpressed in prostate cancer with little expression in peripheral tissue [3]. To further investigate the suitability of this gene as a target for immunotherapy, we used the Oncomine database to examine the expression of SIM2 in other cancers (**Figure 1A**). Our initial findings in prostate cancer were replicated in other prostate cancer datasets within the Oncomine database (**Figure 1B**). Additionally, we found that many other cancers overexpressed SIM2. In particular, colon cancer had more than a 4-fold increase (**Figure 1C**) in SIM2 expression, and breast cancer had more than a 2-fold increase (**Figure 1E**). Significant increases were also found in pancreatic cancer and oligodendroglioma (**Figure 1D and 1F**). Together, these data indicate that SIM2 is an attractive immunotherapeutic target for a wide range of cancers.

### SIM2_237_ is naturally processed and presented on HLA-A*0201 in prostate cancer cells

**Figure 2:**
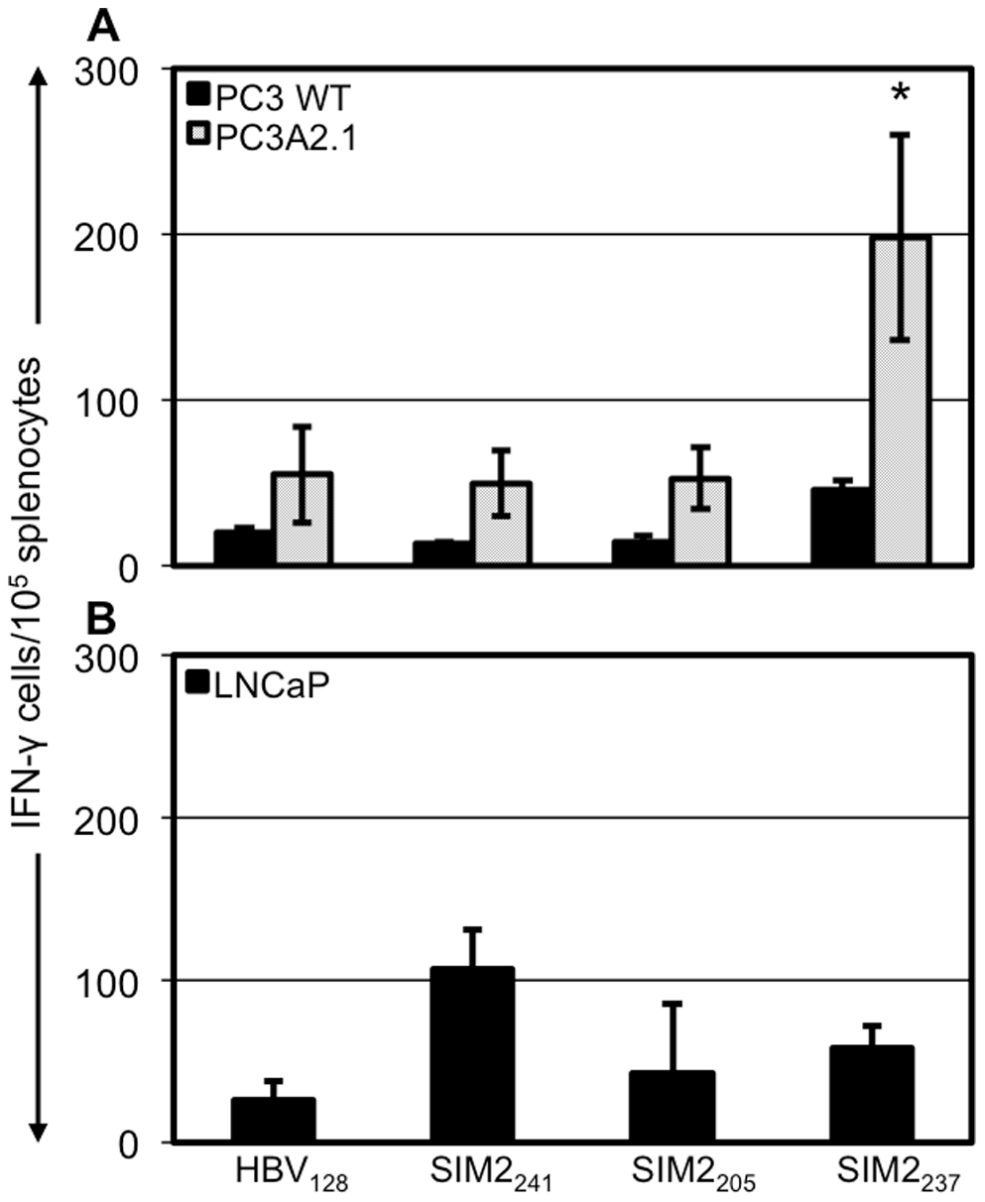
T-cells isolated from SIM2 immunized mice respond to human Prostate Cancer cell lines expressing ERG. Reactivity of splenocytes from SIM2 peptide immunized mice against human prostate cancer cell lines PC3 (**A**) and LNCaP (**B**). Splenocytes from HHD mice immunized with HBV and various SIM2-derived peptides or HBV alone were co-cultured with PC3 or LNCaP. Production of IFN-γ by splenocytes in response to these tumor cell lines was assessed by ELISPOT. Figures show mean ± standard deviation of 3 mice from one experiment. The effect of splenocytes from SIM2-immunized mice against the cell lines was repeated in 2 separate experiments.

Previously, we had identified a number of 9aa long immunogenic HLA-A*0201-restricted epitopes derived from SIM2 [3]. To determine if any of these immunogenic peptides were processed and presented by human prostate cancer cells expressing SIM2, we investigated the activity of splenocytes from HLA-A*0201 transgenic HHD mice immunized with the SIM2_205_ (YQIVGLVAV), SIM2_237_ (SLDLKLIFL), SIM2_241_ (KLIFLDSRV), or control peptide against PC3 and LNCaP cells stably expressing HLA-A*0201. From our previous work, we have identified SIM2 expression in PC3 cells but not LNCaP cells [12]. We found that a significantly increased number of splenocytes isolated from SIM2_237_ produced IFN-γ (198/10^5^ cells) in response to PC3-A2.1 cells compared to control mice (55/10^5^ cells), indicating that SIM2-expressing cells process and present this epitope (**Figure 2A**). In contrast, splenocytes from SIM2_241_ and SIM2_205_ immunized mice had no increased activity against the PC3 cells compared to control immunized mice. Additionally, splenocytes from all SIM2 immunized mice had no increased response against PC3 cells that did not express HLA-A*0201, indicating that this effect was dependent on the MHC-I complex. Splenocyte activity of SIM2 peptide immunized mice was also tested against LNCaP cells, a cell line that does not express SIM2. Splenocytes from all SIM2-immunized mice had no increased activity against these cells compared to controls (**Figure 2B**). These data suggest that SIM2_237_ is the immune-dominant epitope in an HLA-A*0201 restricted setting and could be a potential epitope to target prostate cancer.

### *In silico* design and validation of a multi-epitope vaccine containing the prostate cancer epitope SIM2_237_

**Figure 3:**
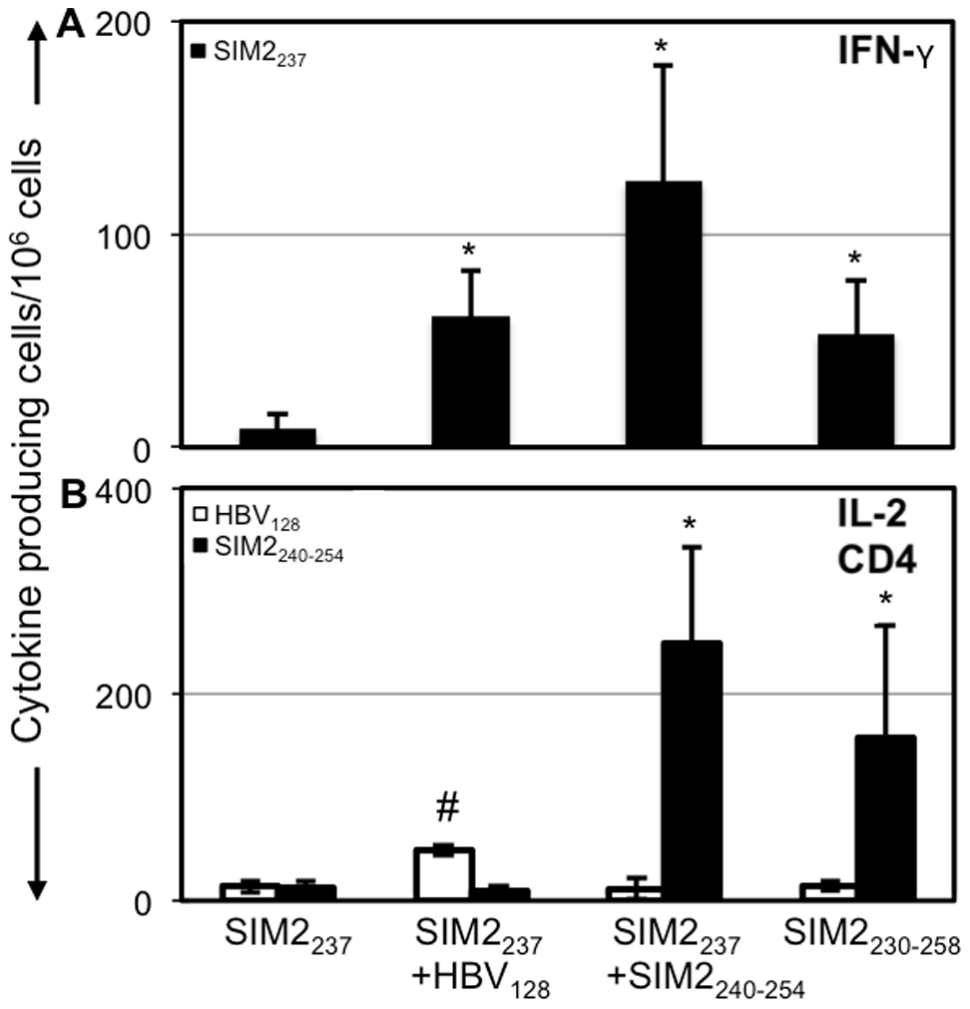
SIM2_230–256_ induces an IFN-γ and CD4 IL-2 response. IFN-γ production by splenocytes in mice immunized with various treatments. Mice were immunized with either the 9aa SIM2_237_ epitope combined with HBV or SIM2_240–254_, or the SIM2_230–256_ peptide alone. IFN-γ production was measured by ELISPOT. IL-2 production by CD4 T-cells. CD4 T-cells were sorted from the spleens of immunized mice and tested for reactivity to HBV_128_ and SIM2_240–254_ by IL-2 ELISPOT.

**Figure 4:**
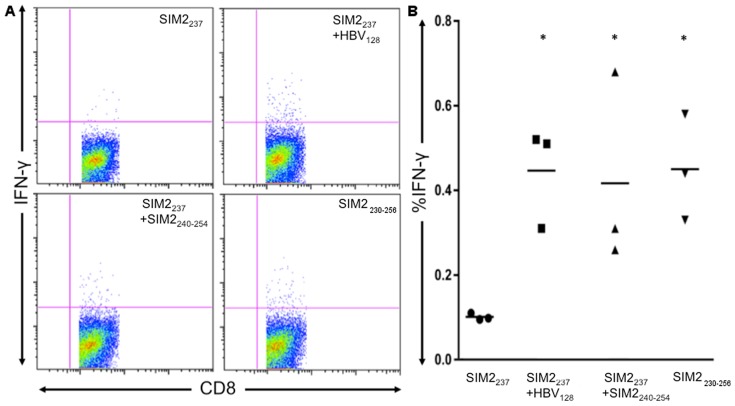
IFN-γ production by CD8 T-cells from SIM2-immunized mice. Mice were immunized with either SIM2_237_, SIM2_237_+HBV_128_, SIM2_237_+SIM2_240–254_ or SIM2_230–256_. Splenocytes were harvested and incubated overnight with T2 cells loaded with the SIM2_237_ peptide. IFN-γ was measured by flow cytometry. FACS plots show the median IFN-γ production for each group (A) and replicate data obtained from each group (B).

While immunization with SIM2_237_ generated an antigen specific response against tumor cells, the immunization regimen required the addition of an HBV-derived I-Ab-restricted epitope (HBV_128_) to induce this response. While providing CD4 stimulation using an HBV-derived peptide is efficient, it does not generate tumor antigen specific CD4 cells. Because CD4 T cells can directly kill tumor cells, expanding cells specific for the target tumor antigen may be beneficial. To eliminate the need of the HBV helper peptide, we hypothesized that CD4 and CD8 T-cells could be stimulated by a single peptide derived from SIM2 containing both MHC-I and MHC-II binding epitopes. To design this multi-epitope long peptide, we extended the amino acids around the SIM2_237_ core and used prediction algorithms to determine if the longer peptides had MHC-II binding potential. Following this approach, we found that this peptide was predicted to bind many human MHC-II molecules (**Table 1**). This peptide also included an I-Ab-restricted epitope, allowing us to test whether the long SIM2 peptide could induce a SIM2_237_ response in the HHD mice. To determine the *in vivo* immunogenicity of the longer peptide, we immunized HHD mice with the SIM2_237_ CD8 epitope and CD4 HBV helper peptide or the long peptide containing both the MHC-I and MHC-II peptide (**Figure 3A**). Mice immunized with SIM2_237_ peptide alone had no significant recall response to the peptide. In contrast, mice immunized with both SIM2_237_ and HBV_128_ had a significantly increased IFN-γ recall response to the SIM2_237_ antigen. We found that replacing the HBV_128_ peptide with the SIM2 derived MHC-II, SIM2_240–254_ (LKLIFLDSRVTEVTG) still generated a recall response to the SIM2_237_ epitope. Similarly, when mice were immunized with SIM2_230–256_ (NMFMFRASLDLKLIFLDSRVTEVTGYE) containing both MHC-I and MHC-II epitopes, a significant CD8 recall response to the SIM2_237_ epitope was elicited. Intracellular flow cytometry for IFN-γ confirmed these findings, showing that splenocytes from mice immunized with either the SIM2_237_+HBV_128_ combination, SIM2_237_+SIM2_240–254_ combination or the longer SIM2_230–256_ peptide all generated a significant IFN-γ recall response to the SIM2_237_ epitope (p<0.05) (**Figure 4**). Additionally, the IL-2 response of CD4 cells to the helper peptides was measured by ELISPOT. Mice immunized with the HBV_128_ generated a significantly greater recall response to HBV_128_ compared to controls. Similarly, mice immunized with SIM2_240–254_ or SIM2_230–256_ generated a significantly greater IL-2 response to the MHC-II-restricted SIM2_240–254_ epitope compared to controls. These data support our hypothesis that the longer SIM2_230–256_ peptide could simultaneously generate both a CD4 IL-2 response against the SIM2_240–254_ epitope, as well as an IFN-γ response against the SIM2_237_ epitope.

**Table 1:** Human MHC-II-restricted epitopes predicted from the SIM2 long peptide using IEDB tool.

Allele	Sequence	Percentile Rank^a^	Comb.Lib. IC50(nM)^b^
HLA-DRB1*07:01	MFMFRASLDLKLIFL	0.31	57.04
HLA-DPA1*02:01/DPB1*01:01	MFMFRASLDLKLIFL	0.92	7
HLA-DRB1*09:01	MFMFRASLDLKLIFL	1.78	1.18
HLA-DPA1*02:01/DPB1*05:01	FMFRASLDLKLIFLD	1.83	0.19
HLA-DRB4*01:01	LDLKLIFLDSRVTEV	1.98	130.98
HLA-DPA1*03:01/DPB1*04:02	MFMFRASLDLKLIFL	2.08	3.31
HLA-DPA1*01/DPB1*04:01	NMFMFRASLDLKLIF	3.22	138.55
HLA-DQA1*01:01/DQB1*05:01	MFMFRASLDLKLIFL	3.91	0.48
HLA-DPA1*01:03/DPB1*02:01	NMFMFRASLDLKLIF	4.69	0.72
HLA-DRB3*01:01	NMFMFRASLDLKLIF	5.31	113.08
HLA-DRB1*01:01	LKLIFLDSRVTEVTG	7.88	0.67
HLA-DQA1*04:01/DQB1*04:02	MFRASLDLKLIFLDS	9.51	190.23
HLA-DQA1*05:01/DQB1*02:01	NMFMFRASLDLKLIF	11.5	2.95
HLA-DQA1*03:01/DQB1*03:02	RASLDLKLIFLDSRV	15.99	58.07

Only the top epitopes having the lowest percentile score and lowest IC50 are selected. One epitope is shown for each HLA allele out of 137 predicted binders.

a Percentile Rank – Percentage of all peptides binding with this efficacy or lower.

b CombLib IC40 – Predicted peptide concentration required to bind 50% of MHC molecules.

### The multi-epitope SIM2_230–256_ peptide induces an antigen-specific response against human SIM2-expressing prostate cancer cell lines

**Figure 5:**
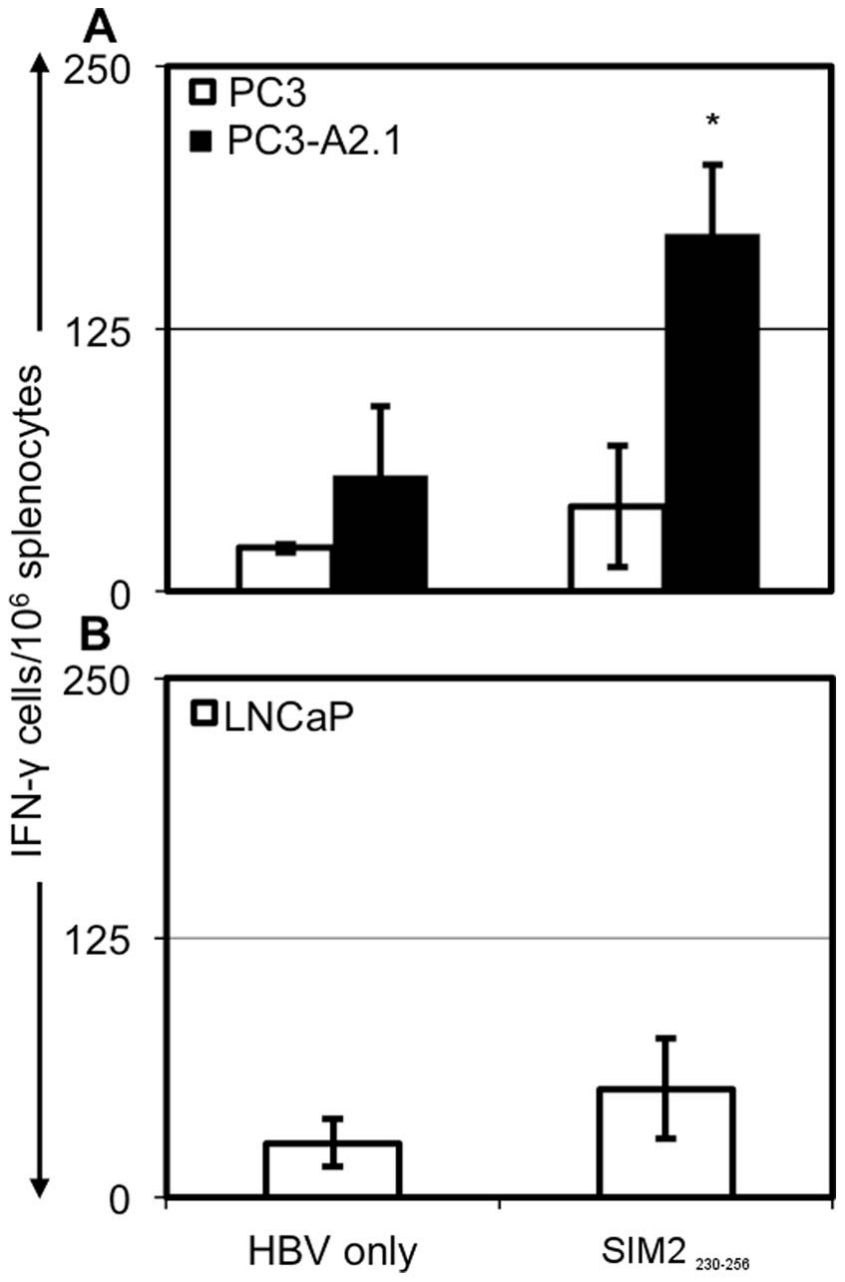
Splenocytes from SIM2_230–256_-immunized mice response to PC3-A2.1 cells. Splenocytes from HHD mice immunized with HBV and various SIM2_230–256_ peptides or HBV alone were co-cultured with PC3, PC3-A2.1 (**A**) or LNCaP (**B**). Production of IFN-γ by splenocytes in response to these tumor cell lines was assessed by ELISPOT. Data is representative of 2 experiments and shows mean ± standard deviation.

We further tested the effectiveness of the SIM2_230–256_ peptide by measuring the IFN-γ recall response against SIM2-expressing prostate cancer cells. HHD mice were immunized with either the long peptide or HBV_128_. Splenocytes were co-cultured with PC3 or PC3-A2.1 cells and recall activity was measured by IFN-γ ELISPOT. Splenocytes isolated from mice immunized with the long-SIM2 peptide had significantly increased activity against PC3-A2.1 cells. Additionally, the number of splenocytes isolated from long-SIM2 immunized mice responding to the PC3-A2.1 cells was significantly higher than those responding to the PC3-WT cell line, indicating that this recall response was dependent on expression of HLA-A*0201 (**Figure 5A**). Cells from SIM2_230–256_-immunized mice were also tested for activity against the SIM2 negative cell line LNCaP. No increased IFN-γ response was detected against this cell line (**Figure 5B**). Together these data suggest that a single peptide containing both MHC-I and MHC-II epitopes derived from SIM2 can induce T-cell activity against SIM2-expressing prostate cancer cells.

## Discussion

Peptide vaccines have been traditionally designed to elicit CTL responses against tumor antigens [18]–[21] resulting in some, but limited clinical benefit, mainly due to the transience and low magnitude of the immune responses they induce [22]. However, recent evidence suggests the importance of CD4 T helper cells in the anti-tumor immune process [23]–[25]. The contribution of CD4 T cells to antigen-specific immunity is well appreciated in mounting immune responses to pathogens, a well-orchestrated process whereby both class I and class II MHC-mediated epitope presentation takes place [26]. Activated CD4 T cells secrete many cytokines that stimulate dendritic cells, leading to enhanced antigen presentation and potentiated anti-tumor immunity [27], [28]. In addition, CD4 T cell-mediated responses are suspected to contribute to the establishment of memory responses [29]. CD4 cells have also been found to develop cytotoxic activity and be able to eradicate melanoma tumors in lymphopenic hosts [30]. Collectively, these findings provide rationale for induction of CD4 T cell responses with cancer vaccines, either alone, or in combination with MHC-I-restricted epitopes.

We and others have previously demonstrated overexpression and specificity of SIM2 in prostate cancer patients [31], [3], [12]. Additionally, we have identified SIM2-derived, HLA-A2.1-restricted epitopes that exhibit the ability to break immune tolerance to SIM2 in mice, and identified SIM2-specific auto-antibodies in sera from patients with PCa [3]. Our work has subsequently suggested a biological role for SIM2 in PCa [12]. However, we have not determined whether the HLA-A2.1-restricted epitopes we identified are naturally processed and presented in tumor cells, nor have we identified longer epitopes that could also trigger CD4 T cell responses. In the present work, we show that overexpression of SIM2 is not limited to PCa. SIM2 is similarly overexpressed in several other malignancies, including colon cancer, breast cancer, cervical cancer, pancreatic cancer and oligodendroglioma, suggesting SIM2 may be an attractive target for immunotherapy of a wide range of cancers. Interestingly, while an overexpression of SIM2 in cancer might suggest a tumorigenic role for SIM2, its frequent down-regulation in other cancers such as oesophageal, kidney, and head and neck cancers (**Figure 1**) might suggest a tumor suppressive role. In fact, SIM2 has been shown to suppress breast cancer growth and invasion in a xenograft model [32]. More intriguing is the observation that Down's syndrome patients are prone to acute leukemia, including acute lymphoblastic leukemia (ALL), while solid tumors, especially breast cancer, is rare [33]. SIM2 is among many transcription factors encoded by genes located on the human chromosome 21. Together, these studies suggest that SIM2 is an attractive immunotherapeutic target for a range of different cancers.

Our data showed that a SIM2_237_-specific response could be elicited against the SIM2-expressing PC3, but not against the SIM2 negative LNCaP cell line (**Figure 2**). However, SIM2_241_ and SIM2_205_ could not induce a CTL response against these same cells, despite both of these peptides showing antigen-specific CTL responses in HLA-A*0201 transgenic mice [3]. In this study we were unable to test the response to another SIM2 expressing prostate cancer cell line, VCaP, due to the cell lines failure to grow after transfection with HLA-A0201. However, The findings that not all immunogenic peptides generate a response against SIM2 expressing cell lines supports the notion that peptide presentation is more complex than MHC-I-binding affinity and that numerous factors contribute to peptide presentation including affinity for the TAP molecule and cytosolic half-life [34], [35]. Nonetheless, together these data indicate that the SIM2_237_ peptide is presented in an HLA-A*0201-restricted manner on cells expressing the SIM2 molecule.

Algorithms that predict MHC-II-restricted epitopes indicate that all proteins, native and mutated, harbor multiple potential MHC-II-restricted epitopes. Compared to MHC-I epitopes, MHC-II-restricted epitopes exhibit a much wider specificity and cross-reactivity, as exemplified by the ability of the PADRE (Pan DR epitope) peptides to recognize a high number of MHC-II alleles in both human and mouse [36]. In the case of SIM2 protein, it is clear many of the epitopes we predicted to bind HLA-DR/DP/DQ would target large populations of patients because of their wide specificity. However, while targeted clinical use of these epitopes would necessitate HLA typing of patients, our mouse immunogenicity tests suggest the long SIM2 peptide harbors an IA-b-restricted epitope(s), as evidenced but the ability of the long peptide to elicit a SIM2_237_-specific CTL response in the absence of the HBV_128_ helper peptide. This response is equal in magnitude to that induced with the combination of HLA-A2.1-restricted SIM2_237_ and the I-Ab-restricted HBV_128_ epitopes. Because long peptides are internalized and processed by dendritic cells, our results indicate a successful internalization and processing of the long peptide and an optimal presentation in the context of both MHC-I and MHC-II complexes to T lymphocytes. The ability of dendritic cells to successfully achieve these steps implies cancer vaccines could be made that contain one single peptide, thus dramatically reducing the cost and regulatory procedures on the path to clinical application. Previous studies have elegantly demonstrated that an increase in the length of the peptide used for vaccination strongly affects the magnitude of the induced CTL response [6], [37]. Comparative experiments showed vaccination with long peptides containing a CTL epitope outperformed vaccination with the CTL peptide alone at inducing effective anti-tumor CTL responses [38]. The low effectiveness of CTL epitopes was shown to be due to the transient nature of the response they can elicit and their failure to induce CTL memory [39].

Together the findings of this study suggest prostate tumor cells expressing SIM2 present the SIM2_237_ epitope in an HLA-A*0201-dependent fashion. Additionally, the multi-epitope peptide SIM2_230–256_ can provide TCR stimulation to both CD4 T cells and CD8 T cells simultaneously. Furthermore, this peptide contains numerous epitopes predicted to bind to various human MHC-II molecules, suggesting that this peptide could induce a CD4 T-cell response in individuals with many different HLA-DR/DP/DQ alleles. Collectively, these data indicate that an effective antigen-specific response can be augmented by concurrent inclusion of class-I and class-II restricted epitopes in peptide vaccine formulations targeting autologous human tumor antigens.
